# Supplementary material for: Growth Profiles of Children and Adolescents Living with and without Perinatal HIV Infection in Southern Africa: A Secondary Analysis of Cohort Data
Source: Nutrients. 2023 Oct 28;15(21):4589. doi: 10.3390/nu15214589 (PMC10650589; doi:10.3390/nu15214589)
Supplement: Supplementary file 1 [file nutrients-15-04589-s001.zip › nutrients-2633090-supplementary.pdf]

Supplementary Tables: Growth profiles of children and adolescents living with and without perinatal HIV infection in Southern Africa: a secondary analysis of cohort data

Table S1. Multinomial logistic multivariable regression showing HIV-related factors independently associated with growth profiles among those with HIV, n=639.

**HIV-related factors**	Profile 2: Tall not thin	Profile 3: Short not thin	Profile 4: Stunted not thin	Profile 5: Thin not stunted	Profile 6: Thin and stunted	Profile 7: Very thin and stunted
	**RRR (95% CI)**	**RRR (95% CI)**	**RRR (95% CI)**	**RRR (95% CI)**	**RRR (95% CI)**	**RRR (95% CI)**
Age at ART initiation, years						
<2	Ref.	Ref.	Ref.	Ref.	Ref.	Ref.
2 to <5	1.19 (0.17, 8.43)	0.36 (0.09, 1.38)	0.83 (0.40, 1.75)	0.99 (0.34, 2.85)	1.24 (0.49, 3.16)	0.56 (0.12, 2.54)
5 to <10	0.90 (0.10, 8.20)	0.34 (0.09, 1.31)	0.86 (0.39, 1.90)	0.67 (0.22, 2.10)	1.43 (0.54, 3.77)	0.46 (0.10, 2.02)
10 or later	1.83 (0.14, 23.3)	0.37 (0.08, 1.73)	0.78 (0.29, 2.14)	0.58 (0.15, 2.22)	1.64 (0.52, 5.14)	0.63 (0.12, 3.20)
HIV viral load copies/ml						
<1000	Ref.	Ref.	Ref.	Ref.	Ref.	Ref.
≥1000	0.64 (0.14, 3.03)	0.58 (0.22, 1.52)	0.95 (0.54, 1.69)	0.57 (0.27, 1.23)	1.23 (0.67, 2.26)	1.63 (0.70, 3.77)
CD4 cell count cells/μL						
≥500	Ref.	Ref.	Ref.	Ref.	Ref.	Ref.
<500	2.63 (0.60, 11.5)	2.20 (0.87, 5.60)	1.39 (0.76, 2.54)	1.08 (0.50, 2.35)	0.85 (0.44, 1.64)	1.51 (0.64, 3.58)

* The reference profile is Profile 1: Average growth. Model was also adjusted for sex, age group, chronic lung disease status, and country of residence. RRR – relative risk ratio.

Table S2. Trunk fat percentage for each latent class profile by sex among IMVASK participants.

Growth profile	Sex	Total in profile	Baseline trunk fat %, mean (SD)	Follow-up trunk fat %, mean (SD)	Change in trunk fat %, mean (SD)
**1. Average growth**	Females	107	22.7 (3.9)	23.1 (3.8)	0.82 (3.51)
	Males	98	17.7 (2.5)	17.8 (2.6)	-0.06 (2.58)
**2: Taller not thin**	Females	40	29.6 (6.4)	29.1 (6.7)	-0.03 (3.49)
	Males	23	22.5 (7.3)	21.4 (5.9)	-0.01 (3.21)
**3: Short not thin**	Females	30	31.9 (7.2)	33.2 (5.7)	1.56 (4.05)
	Males	11	19.7 (4.1)	18.3 (3.9)	-1.21 (4.19)
**4: Stunted not thin**	Females	64	22.9 (5.4)	23.1 (5.2)	0.35 (3.37)
	Males	84	17.6 (2.8)	17.6 (2.5)	-0.02 (2.41)
**5: Thin not stunted**	Females	25	19.1 (2.8)	21.2 (3.7)	2.38 (3.37)
	Males	27	17.8 (3.2)	16.8 (2.7)	-0.41 (1.99)
**6: Thin and stunted**	Females	15	21.0 (5.2)	20.7 (3.4)	-0.23 (2.04)
	Males	38	17.6 (3.4)	17.4 (3.1)	-0.35 (2.80)
**7: Very thin and stunted**	Females	6	25.1 (10.7)	26.0 (9.8)	-0.04 (2.57)
	Males	7	17.4 (2.6)	18.5 (2.3)	0.93 (1.24)

Table S3. Food consumption for IMVASK participants in each latent class profile.

		Consumed food more than 1-2 times per week				
**Growth profile**	**Total in profile**	**Legumes, n (%)**	**Dairy, n (%)**	**Meat, n (%)**	**Eggs, n (%)**	**Fish, n (%)**
**1. Average growth**	205	187 (86.2)	186 (85.7)	210 (96.8)	187 (86.2)	151 (69.6)
**2: Taller not thin**	63	60 (92.3)	60 (92.3)	65 (100)	56 (86.1)	50 (76.9)
**3: Short not thin**	41	37 (90.2)	39 (95.1)	40 (97.6)	39 (95.1)	27 (65.9)
**4: Stunted not thin**	148	139 (89.1)	132 (84.6)	151 (96.8)	132 (84.6)	113 (72.4)
**5: Thin not stunted**	52	48 (81.4)	48 (81.4)	57 (96.6)	51 (86.4)	35 (59.3)
**6: Thin and stunted**	53	49 (87.5)	44 (78.6)	53 (94.6)	46 (82.1)	43 (76.8)
**7: Very thin and stunted**	13	14 (93.3)	10 (66.7)	14 (93.3)	13 (86.7)	9 (60.0)
